# Lymphovascular invasion is an independent prognostic factor in breast cancer irrespective of axillary node metastasis and molecular subtypes

**DOI:** 10.3389/fonc.2023.1269971

**Published:** 2023-11-17

**Authors:** Suk Jun Lee, Jieon Go, Byung Soo Ahn, Jee Hyun Ahn, Jee Ye Kim, Hyung Seok Park, Seung Il Kim, Byeong-Woo Park, Seho Park

**Affiliations:** ^1^ Division of Breast Surgery, Department of Surgery, Yonsei University College of Medicine, Seoul, Republic of Korea; ^2^ Department of Surgery, Eunpyeong St. Mary’s Hospital, The Catholic University of Korea, Seoul, Republic of Korea; ^3^ Department of Pathology, Severance Hospital, Yonsei University, College of Medicine, Seoul, Republic of Korea

**Keywords:** lymphovascular invasion, breast cancer subtypes, node metastasis, oncotype Dx, breast cancer

## Abstract

**Purpose:**

Lymphovascular invasion (LVI) is a well-known poor prognostic factor for early breast cancer. However, the effect of LVI on breast cancer subtype and node status remains unknown. In this study, we aimed to evaluate the clinical significance of LVI on the recurrence and long-term survival of patients with early breast cancer by comparing groups according to the subtype and node status.

**Methods:**

We retrospectively reviewed the medical records of 4554 patients with breast cancer who underwent breast cancer surgery between January 2010 and December 2017. The primary endpoints were disease-free survival (DFS) and overall survival (OS). Univariate and multivariate analyses were performed to identify prognostic factors related to the DFS and OS according to the nodal status and breast cancer subtype.

**Results:**

During a follow-up period of 94 months, the median OS and DFS were 92 and 90 months, respectively. The LVI expression rate was 8.4%. LVI had a negative impact on the DFS and OS, regardless of the lymph node status. LVI was associated with higher recurrence and lower survival in the luminal A, human epidermal growth factor receptor 2-positive, and triple-negative breast cancer subtypes. The Cox proportional hazards model showed that LVI was a significant prognostic factor for both DFS and OS. No correlation has been observed between LVI and the Oncotype Dx results in terms of prognostic value in early breast cancer.

**Conclusion:**

LVI is an independent poor prognostic factor in patients with early breast cancer, regardless of the node status and molecular subtype. Therefore, the LVI status should be considered when making treatment decisions for patients with early stage breast cancer; however, further prospective studies are warranted.

## 1 Introduction

Breast cancer is the most common cancer diagnosed in women worldwide (1). The total number of patients with breast cancer in South Korea has doubled over the last decade (2). Over the years, many studies have identified prognostic factors in breast cancer, such as age, tumor size, axillary lymph node status, histologic grade, estrogen/progesterone receptor (ER/PR) status, human epidermal growth factor receptor 2 (HER2), and Ki-67, which are significant factors that should be considered when deciding on adjuvant treatments (3, 4).

Lymphovascular invasion (LVI) is associated with the recurrence of solid tumors, including early breast cancer (5). However, according to the National Comprehensive Cancer Network (NCCN), St. Gallen, and European Society for Medical Oncology (ESMO) recommendations, sole LVI status has very limited role in deciding adjuvant treatment (6–8). Although genomic assays are widely employed for making decisions regarding adjuvant chemotherapy, LVI can also be an important factor regardless of gene expression status in patients with ER-positive/HER2-negative breast cancer (9). Recent studies have demonstrated that the detection of LVI supplements reliable information to the 21-gene recurrence score (RS) (10, 11).

However, few studies have investigated the correlation between LVI and recurrence and survival according to the molecular subtypes. Therefore, this study aimed to evaluate the prognostic significance of LVI on the recurrence and long-term survival according to the molecular subtype in patients with early breast cancer who underwent breast surgery.

## 2 Materials and methods

### 2.1 Study population

**Figure 1:**
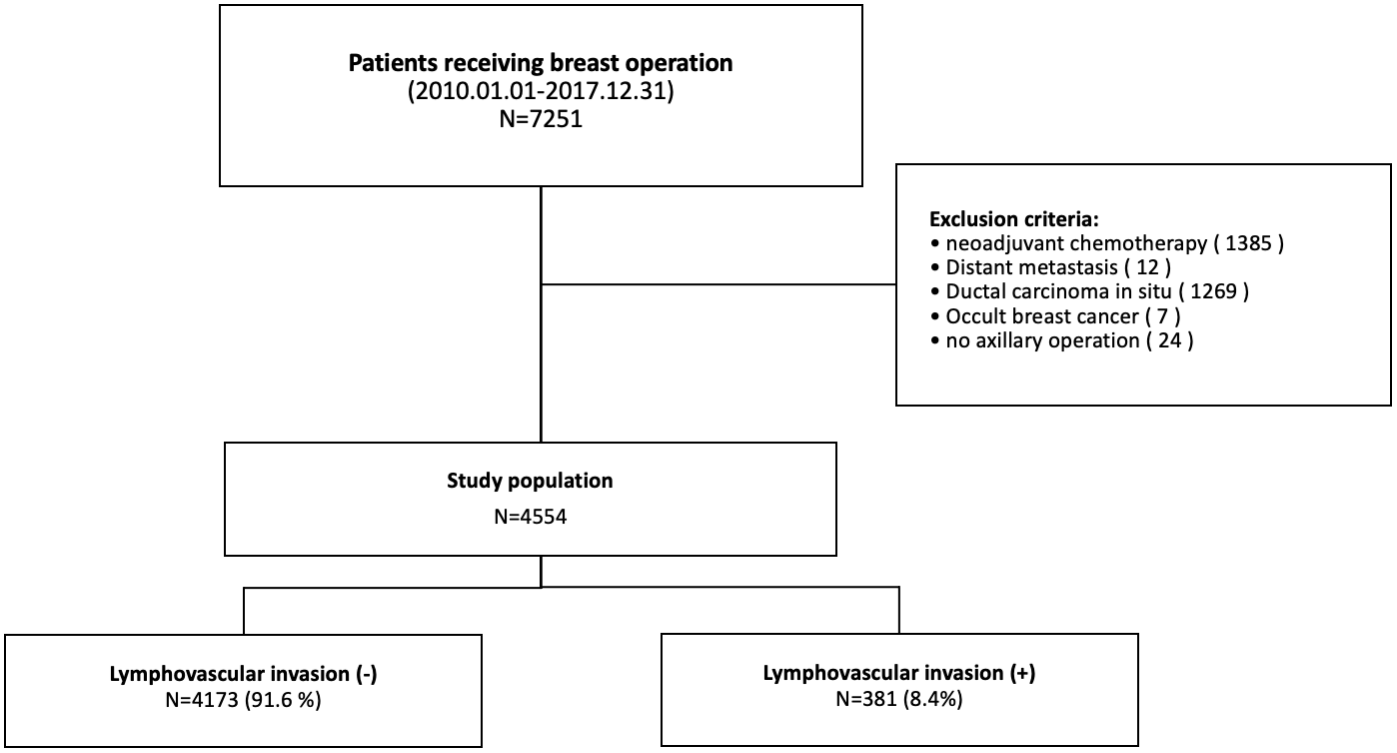
Patient selection flow chart.

We retrospectively reviewed the data of patients with breast cancer who underwent breast cancer surgery at Severance Hospital, Yonsei University College of Medicine, Seoul, South Korea, between January 2010 and December 2017. Patients who received neoadjuvant chemotherapy, presented with distant metastases at diagnosis, were diagnosed with ductal carcinoma *in situ* or occult breast cancer, or did not undergo axillary surgery were excluded from the study. Finally, 4,554 patients were included in the analysis (**Figure 1**).

Survival data were obtained from the medical records of Severance Hospital. This study was approved by the Institutional Review Board of our institution (Approval No. 4-2023-0067), and the requirement for informed consent was waived owing to the retrospective study design.

### 2.2 Clinicopathologic evaluation

Basic patient information, such as age and clinicopathological characteristics, including tumor stage, node stage, histologic grade, nuclear grade, ER status, PR status, HER2 status, Ki-67 index value, adjuvant treatments, radiotherapy, breast surgery type, and axillary surgery type, were collected. Tumor stage was evaluated according to the 8^th^ American Joint Committee on Cancer (AJCC) TNM staging system (12).

**Figure 2:**
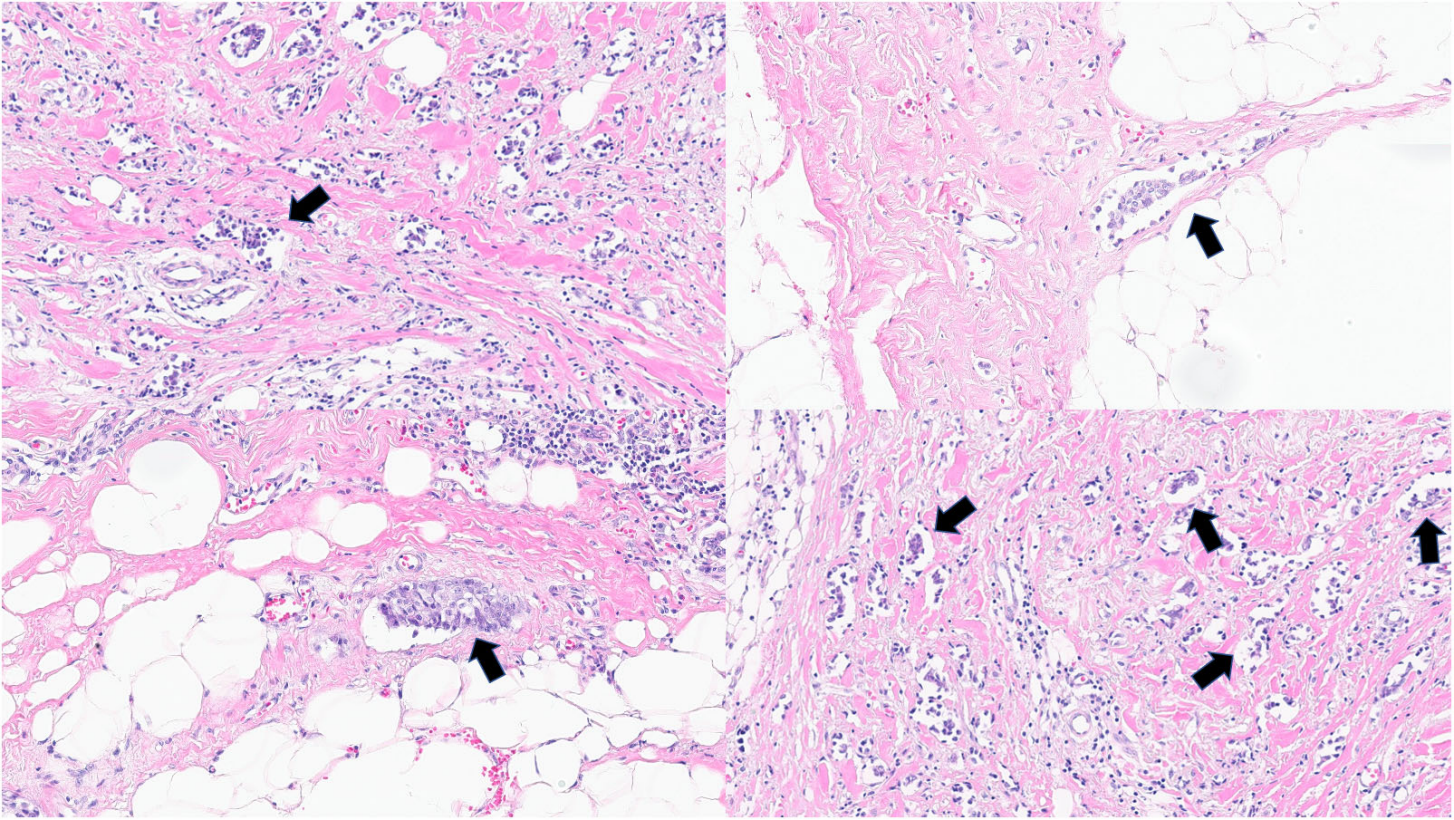
Lymphovascular invasion in invasive breast cancer in hematoxylin and eosin slides. Arrows show lymphovascular invasion. (all magnification, 100×).

The surgical specimens were stained with hematoxylin and eosin (H&E) to identify LVI and reported in routine pathology reports. Lymphatic invasion was defined as the presence of tumor emboli within the endothelial line space, whereas vessel invasion was defined as the presence of fibrin clots or erythrocytes and a lack of smooth muscle or elastic fibers in the endothelial line space. Because immunohistochemical staining is not routinely performed, distinguishing between lymphatic and vessel invasion using light microscopy was challenging. Thus, in our study, LVI was defined as the presence of tumor cells within the endothelial line spaces around the tumor (**Figure 2**).

Breast cancer molecular subtypes were defined based on the immunohistochemical staining results as follows: a) hormone receptor (HRs)-positive (ER/PR) and HER2 negative tumors, b) HRs positive and HER2 positive tumors, c) HRs negative and HER2 positive tumors, and d) tumors negative for both HRs and HER2, defined as triple-negative breast cancer (TNBC). According to the guidelines of the American Society of Clinical Oncology (ASCO) 2010, the positive status of ER or PR is defined as the presence of at least 1% stained cancer nuclei of ER or PR. HER2 positivity was defined as a score of 3 on immunohistochemical analysis (13, 14). HER2 expression of 0 or 1+ were categorized as HER2 negative, and HER2 expression of 3+ was defined as HER2 positive. If HER2 expression was 2+, the silver *in situ* hybridization assay was performed. Positive silver *in situ* hybridization assay results were categorized as HER2 positive, and vice versa. ≥ 20% Ki‐67 index values were classified as highly proliferative tumors.

### 2.3 Statistical analysis

Recurrence was defined as recurrence in the ipsilateral breast or counterlateral breast, regional or non-regional lymph node areas, or distant organs. Disease-free survival (DFS) was defined as the time from diagnosis to disease recurrence or death, whichever occurred first. Overall survival (OS) was defined as the time from diagnosis to death from any cause.

Categorical factors were analyzed using the chi-square test. The Kaplan–Meier method was used to draw DFS and OS curves, and group differences were calculated using the log-rank test. A multivariate Cox proportional hazards model was used to identify significant independent factors associated with DFS and OS. Statistical significance was defined as *p* < 0.05. All the statistical analyses were performed using SPSS software (version 26.0; IBM Software, Armonk, NY, USA).

## 3 Results

### 3.1 Patient characteristics

LVI was observed in 381 patients (8.4%). Comparisons of the clinicopathological characteristics of the patients with and without LVI are shown in **Table 1**. The mean age of the patients in the LVI-negative group was higher than that of the patients in the LVI-positive group. Patients with positive LVI showed higher stages and grades than those with negative LVI. Specifically, a higher N stage was associated with a higher LVI positivity rate. The LVI positivity rates were 15.4%, 19.6%, 31.9%, and 52.5% in the pN1mi, N1, N2, and N3 stages, respectively.

**Table 1:** Patient characteristics according to the lymphovascular invasion status.

	Lymphovascular invasion status				
	Negative (N=4173, 91.6%)		Positive (N=381, 8.4%)		*p*-value
	N	%	N	%	
Age, years					<0.001
<50	1905	89.9	213	10.1	
≥50	2268	93.1	168	6.9
T stage					<0.001
1	3268	94.0	210	6.0	
2	871	84.6	159	15.4
3	34	73.9	12	26.1
N stage					<0.001
N0	3450	95.2	174	4.8	
N1mi	181	84.2	34	15.8
N1	432	80.6	104	19.4
N2	82	68.3	38	31.7
N3	28	47.5	31	52.5
Histologic Grade					<0.001
1	1151	96.6	40	3.4	
2	2118	89.9	233	10.1
3	904	89.1	108	10.9
Ki-67					0.033
<20%	2868	92.3	239	7.7	
≥20%	1224	90.4	130	9.6
Subtypes					0.01
HRs(+) HER2(-)	2448	91.8	198	8.2	
HRs(+) HER2(+)	846	88.0	115	12.0
HRs(-) HER2(+)	326	91.1	32	8.9
HRs(-) HER2(-)	553	93.9	36	6.1
Endocrine therapy					0.283
No	933	92.2	79	7.8	
Yes	3207	91.4	302	8.6	
Herceptin treatment					<0.001
No	3639	92.5	293	7.5	
Yes	466	86.9	70	13.1	
Adjuvant Chemotherapy					<0.001
No	2038	96.3	79	3.7	
Yes	2135	87.5	305	12.5
Radiotherapy					0.011
No	1314	93.2	96	6.8	
Yes	2859	90.9	285	9.1
Breast operation					<0.001
PM	2643	93.0	199	7.0	
TM	1530	89.4	182	10.6
Axillary operation					<0.001
SLNB	3504	94.8	193	5.2	
SLNB+ALND	669	78.1	188	21.9

HRs, hormone receptor; HER2, human epidermal growth factor-2 receptor; PM, partial mastectomy; TM, total mastectomy; SLNB, sentinel lymph node biopsy; ALND, axillary lymph node dissection.

In addition, the LVI-positive group had a higher percentage of patients with high Ki-67 levels than the LVI-negative group. Regarding the molecular subtypes, the LVI-positive group had a lower percentage of luminal type but a higher proportion of HER2 positive and TNBC subtypes. The percentage of patients who received adjuvant chemotherapy and radiotherapy was higher in the LVI-positive group than that in the LVI-negative group (*p*=0.011). The LVI-positive group underwent more total mastectomies and axillary lymph node dissections than the LVI-negative group.

### 3.2 Survival outcomes and prognostic factors

**Figure 3:**
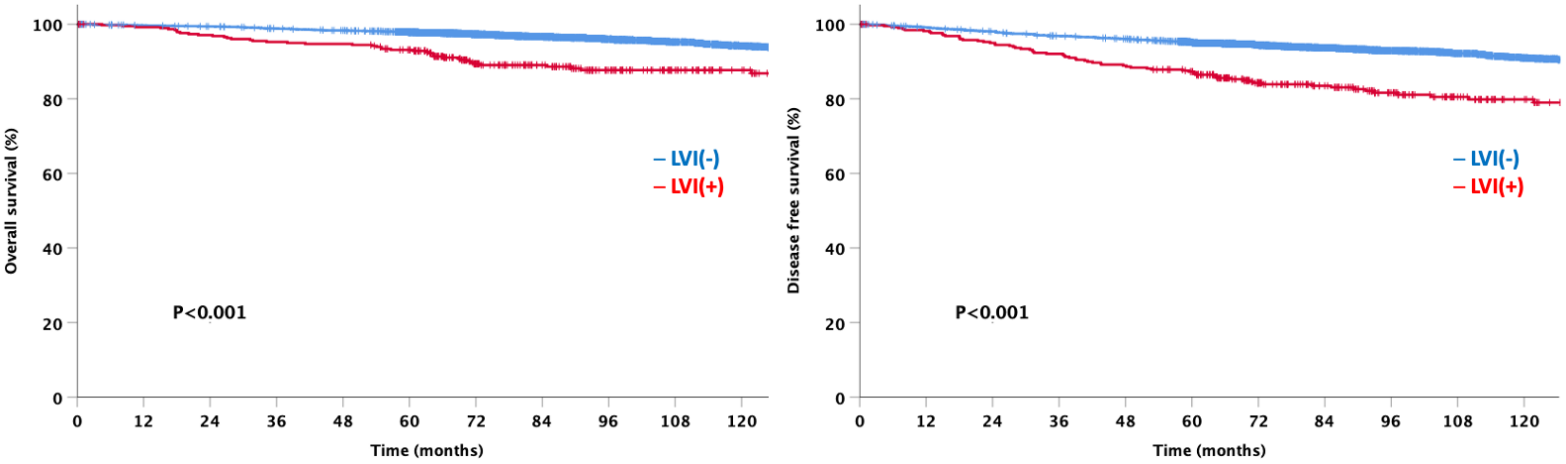
Overall and disease-free survival according to lymphovascular invasion status.

**Figure 4:**
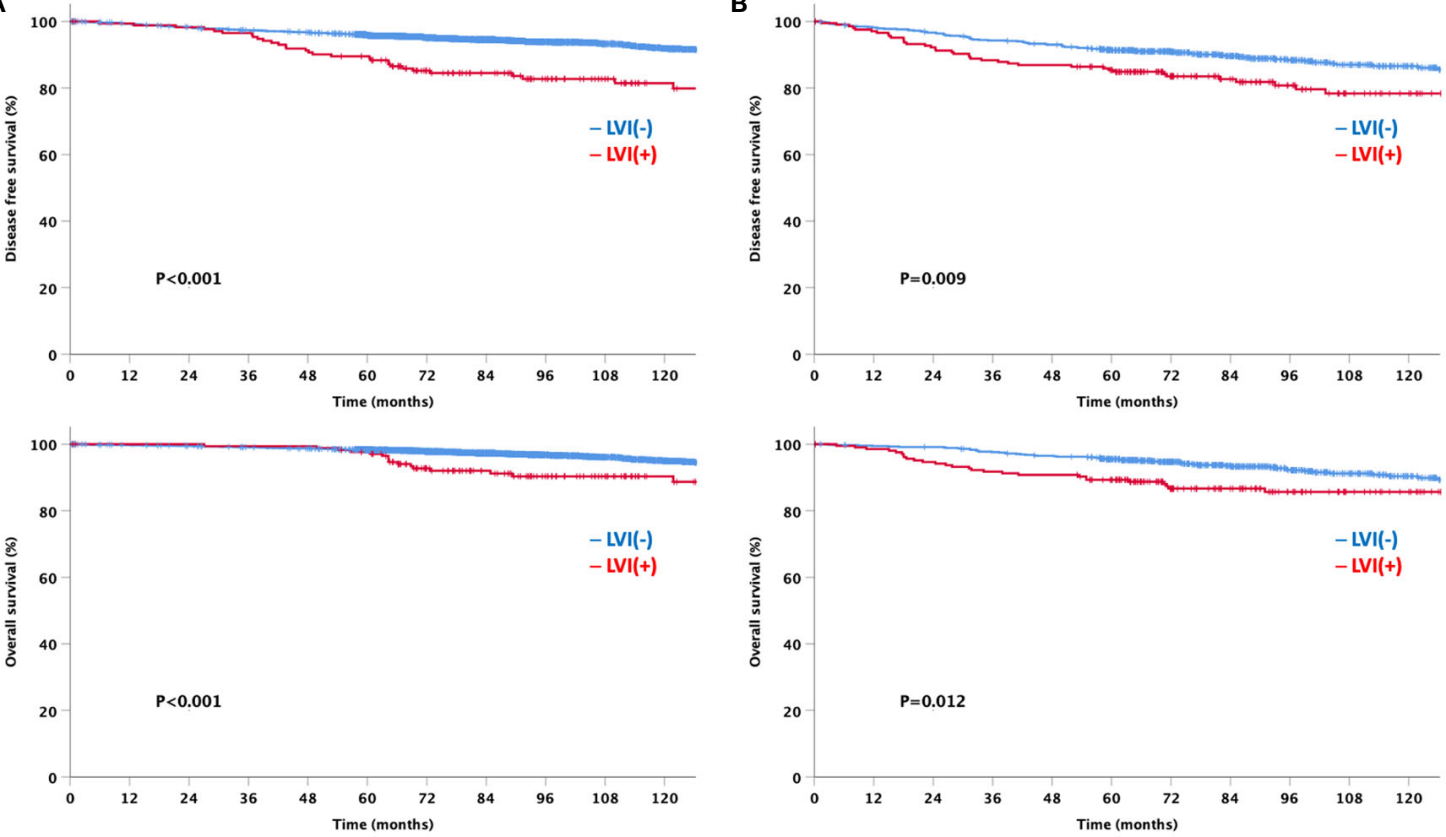
Disease-free and overall survival based on the lymphovascular invasion according to node status **(A)** Node negative **(B)** Node positive.

**Figure 5:**
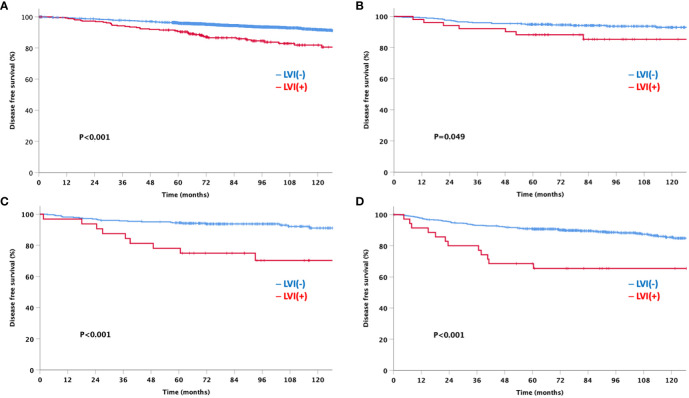
Disease-free survival by lymphovascular invasion according to the breast cancer subtype **(A)** HRs+HER2- **(B)** HRs+HER2+ **(C)** HRs-HER2+ **(D)** HRs-HER2-HRs: hormone receptor, HER2: human epidermal growth factor receptor 2.

At a median follow-up of 92 months, the LVI-negative group showed significantly favorable DFS and OS compared to the LVI-positive group (**Figure 3**). In addition, a significant difference was observed in the DFS between the two groups for both node-negative and node-positive disease (**Figure 4**). Moreover, the LVI-positive group showed a poorer prognosis than the LVI-negative group, regardless of the breast cancer subtype (**Figure 5**).

The univariate analyses associated with DFS are presented in **Table 2**. Older age, higher T stage, higher N stage, higher histological grade, positive LVI, TNBC subtype, adjuvant chemotherapy, endocrine therapy, or radiotherapy, and total mastectomy were significant prognostic factors associated with poor DFS. In the multivariate analysis, higher T stage, higher N stage, higher histologic grade, positive LVI, and radiotherapy were statistically significant.

**Table 2:** Prognostic factors for overall survival and disease-free survival associated with lymphovascular invasion.

		Disease Free Survival								Overall Survival							
		Univariate				Multivariate				Univariate				Multivariate			
		HR	95% CI		*p*-value	HR	95% CI		*p*-value	HR	95% CI		*p*-value	HR	95% CI		*p*-value
Age	≥50 vs. <50	1.286	1.044	1.583	0.018	1.208	0.972	1.502	0.088	1.926	1.455	2.550	<0.001	1.781	1.328	2.387	<0.001
T stage	≥2 vs. 1	2.264	1.839	2.788	<0.001	1.618	1.275	2.054	<0.001	2.288	1.754	2.985	<0.001	1.616	1.192	2.192	0.002
N stage	0	ref				ref				ref				ref			
	1	1.633	1.271	2.099	<0.001	1.399	1.034	1.893	0.029	1.920	1.407	2.662	<0.001	1.846	1.268	2.688	0.001
	2–3	3.729	2.670	5.209	<0.001	2.898	1.808	4.644	<0.001	4.314	2.858	6.511	<0.001	3.943	2.231	6.971	<0.001
Histologic Grade	1	ref				ref				ref				ref			
	2	1.784	1.327	2.397	<0.001	1.435	1.050	1.960	0.023	1.758	1.198	2.580	0.004	1.543	1.03	2.312	0.035
	3	2.563	1.869	3.515	<0.001	1.850	1.256	2.724	0.002	2.713	1.812	4.062	<0.001	2.322	1.42	3.798	0.001
LVI	(+) vs (-)	2.563	1.966	3.339	<0.001	1.766	1.301	2.397	<0.001	2.744	1.973	3.815	<0.001	1.958	1.344	2.853	<0.001
Subtype	HRs(+) HER2(-)	ref				ref				ref				ref			
	HRs(+) HER2(+)	0.994	0.683	1.446	0.973	0.871	0.497	1.528	0.630	0.748	0.440	1.273	0.285	0.448	0.187	1.075	0.072
	HRs(-) HER2 (+)	1.258	0.869	1.821	0.224	1.137	0.502	2.573	0.759	0.914	0.537	1.555	0.740	0.432	0.144	1.297	0.135
	HRs(-) HER2(-)	1.830	1.412	2.372	<0.001	1.845	0.953	3.569	0.069	1.819	1.315	2.514	<0.001	1.315	0.599	2.888	0.495
Adjuvant Chemotherapy	Yes vs. No	1.503	1.214	1.862	<0.001	0.912	0.692	1.201	0.510	1.223	0.942	1.614	0.127	0.662	0.468	0.937	0.02
Endocrine therapy	Yes vs. No	0.614	0.493	0.765	<0.001	1.140	0.608	2.138	0.684	0.624	0.471	0.827	0.001	0.825	0.392	1.735	0.612
Herceptin treatment	Yes vs. No	1.032	0.750	1.420	0.846	0.843	0.478	1.486	0.554	0.849	0.546	1.320	0.849	1.287	0.545	3.039	0.566
Radiotherapy	Yes vs. No	0.570	0.464	0.701	<0.001	0.581	0.387	0.871	0.009	0.571	0.438	0.743	<0.001	0.575	0.352	0.941	0.028
Breast surgery	TM vs. PM	2.199	1.790	2.702	<0.001	1.204	0.807	1.797	0.362	2.242	1.721	2.921	<0.001	1.222	0.751	1.99	0.420

LVI, lymphovascular invasion; HRs, hormone receptor; HER2, human epidermal growth factor-2 receptor; TM, total mastectomy; PM, partial mastectomy; CI, confidence interval.

Prognostic factors associated with OS are shown in **Table 2**. In the univariate analysis, age of ≥ 50 years, larger tumor size, advanced nodal stage, grade III tumors, presence of LVI, TNBC subtype, endocrine therapy or radiotherapy, and total mastectomy were worse prognostic factors related to the OS. Multivariate analyses revealed that older age, higher T stage, higher N stage, higher histologic grade, positive LVI, adjuvant chemotherapy, and radiotherapy were significant independent prognostic factors.

### 3.3 Association between multigene assay and LVI

**Figure 6:**
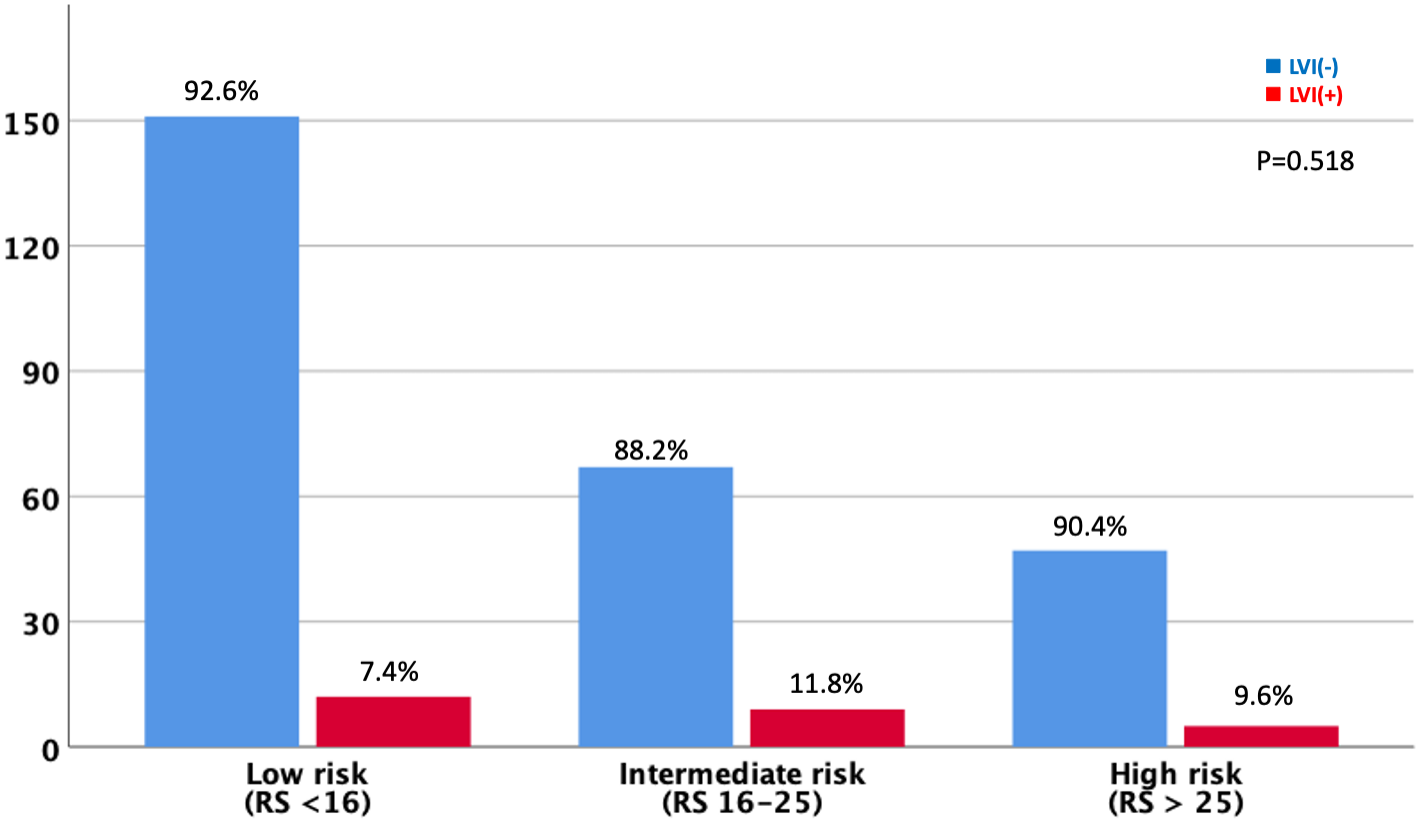
Lymphovascular invasion expression based on the Oncotype Dx recurrence scores.

**Figure 7:**
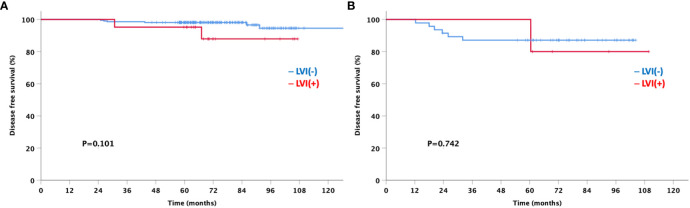
Disease-free survival based on the lymphovascular invasion according to Oncotype Dx group **(A)** low/intermediate-risk **(B)** high-risk.

Of the 4,554 patients included in the analysis, only 291 were available for the Oncotype Dx 21-gene recurrence score (RS) to explore the association between the multigene assay and the anatomical presence of LVI in patients with HR-positive and HER2-negative tumors. Patients were classified into three groups based on the RS according to the TAILORx trial (15); 163 (56.0%), 76 (26.1%), and 52 (17.9%) patients were in the low risk (RS ≤15), intermediate risk (RS 16–25), and high risk (RS ≥26) groups, respectively (**Figure 6**). No significant difference was observed in the prevalence of LVI among the three groups (7.4%, 11.8%, and 9.6% in the low-, intermediate-, and high-risk groups, respectively; *p*=0.518). Although a limited number of patients underwent the 21-gene RS assay and had a favorable luminal subtype, we further analyzed the DFS to explore the impact of the presence of LVI on the survival outcomes according to the Oncotype Dx risk classification. Patients with positive LVI showed a trend towards worse DFS; however, the difference was not statistically significant regardless of the 21-gene RS risk classification (**Figure 7**). In addition, the OS of LVI-positive patients was significantly worse in the Oncotype Dx low-/intermediate-risk group, whereas no difference in the OS was observed in the Oncotype Dx high-risk group. However, the actual events were too rare for observing any statistical significance in the current analysis.

## 4 Discussion

This study demonstrated the clinical significance of LVI in early breast cancer in a large population with a long 10-year follow-up period. The detection rate of LVI was 8.4% in our study, which is consistent with previous studies (10, 16, 17), but lower than some studies reporting up to 30–48% (18, 19). The retrospective design and mere review of routine pathology medical records could be attributed to the difference. The race, limited to the Asian population, may be another reason for this. We assumed that a large proportion of smaller T1 stages in our data may have affected the low percentage of LVI expression.

Our study demonstrated that LVI is an independent poor prognostic factor for survival in patients with early breast cancer. Whether LVI should be considered an independent prognostic factor is debatable. While some studies have suggested that LVI is an independent prognostic variable not affected by the lymph node status or pathological features (20), other studies have stated that LVI is the only dependent factor associated with tumor characteristics, such as histological grade (21, 22). Some studies have even stated that LVI is not related to the treatment outcomes in patients with breast cancer (23, 24). Nevertheless, very few studies have analyzed LVI in the context of node status and molecular subtypes. In this study, we observed that LVI had a negative impact on the DFS and OS, regardless of the node status. Furthermore, LVI is associated with higher recurrence and lower survival rates in all breast cancer subtypes. Moreover, LVI was associated with DFS and OS in both univariate and multivariate analyses. These results suggest that LVI has independent prognostic value in patients with early breast cancer.

According to the findings of this study, while radiation therapy status was not statistically different between LVI negative and positive groups, radiation therapy was also an independent prognostic factor for overall survival. Since postmastectomy radiation therapy is administered for patients with risk factors such as large tumor size, close or positive resection margin, high histologic grade, and hormone receptor negative tumors, such factors might have affected the overall prognosis (6). The St. Gallen International Consensus Guidelines also recommend whole breast irradiation over partial breast irradiation in patients with LVI positive tumors (25).

Our study demonstrated the prognostic power of LVI; however, discordant findings were observed between LVI and node metastasis. Our data showed that 8.7% of the patients were LVI-positive, while 20.4% were node-positive. In the subgroups, node negative disease (N0) was 82.7% in the LVI-negative group and 54.3% in the LVI-positive group. Although the presence of LVI increased as the N stage increased (pN1mi,15.4%; N1,19.6%; N2,31.9%; and N3,52.5%), LVI was not a prerequisite condition for axillary node metastasis. Previous studies have also stated that lymph node metastasis can occur even in the absence of LVI in breast cancer (26). Thus, lymph node metastasis is not completely preventable because LVI does not occur. Taken together, our results are consistent with those of previous studies comparing LVI and node metastasis.

Furthermore, no association was observed between LVI and Oncotype Dx, indicating its prognostic value in early breast cancer. This finding is in agreement with a study by Al-Zawi et al., which stated that LVI did not have a statistically significant impact on the Oncotype Dx RS (9). Recent studies have shown that the detection of LVI adds reliable information to the 21-gene RS (10, 11). LVI provides additional prognostic information for OS in N0 patients with RS of 11-100 (10). In addition, one study investigated the genes used for multi-gene assays in LVI-positive patients. In the study, the authors found out that the Oncotype Dx test generally focuses on genes about proliferation, HER2 status, estrogen receptor and invasion, and only one out of twenty-one ODX gene correlated with LVI (27). Thus, we suggest that LVI should be considered a significant prognostic factor for early stage breast cancer, regardless of the genomic assay results.

Our study had several limitations, mostly owing to its retrospective study design. Second, the rate of LVI expression was slightly lower than that reported in other studies. This is because a smaller tumor size could have affected the results of LVI detection. Third, Oncotype Dx result was only available for small portion of study population. Therefore, we cannot perform powerful analysis about LVI and oncotype Dx result. Finally, the possibility of selection bias cannot be excluded. Despite these limitations, our study had a long follow-up period of up to 140 months and more than 4,000 patients were included in the analysis.

In conclusion, LVI in patients with early breast cancer is an independent risk factor for poor prognosis. Patients with positive LVI showed a higher recurrence rate and poorer survival in all breast cancer subtypes. Therefore, the LVI status should be considered when making treatment decisions for patients with early breast cancer; however, further prospective studies are warranted.

## Data availability statement

The raw data supporting the conclusions of this article will be made available by the authors, without undue reservation.

## Ethics statement

The studies involving humans were approved by Institutional Review Board of Severance Hospital, Seoul, Republic of Korea (Approval No. 4-2023-0067). The studies were conducted in accordance with the local legislation and institutional requirements. The ethics committee/institutional review board waived the requirement of written informed consent for participation from the participants or the participants’ legal guardians/next of kin owing to the retrospective study design.

## Author contributions

SL: Writing – original draft, Writing – review & editing. JG: Validation, Writing – review & editing. BA: Data curation, Writing – review & editing. JA: Data curation, Writing – review & editing. JK: Data curation, Writing – review & editing. HP: Data curation, Writing – review & editing. SK: Writing – review & editing. B-WP: Writing – review & editing. SP: Data curation, Formal analysis, Supervision, Validation, Writing – review & editing.
