# Supplementary material for: Lymphovascular invasion is an independent prognostic factor in breast cancer irrespective of axillary node metastasis and molecular subtypes
Source: Front Oncol. 2023 Nov 17;13:1269971. doi: 10.3389/fonc.2023.1269971 (PMC10694501; doi:10.3389/fonc.2023.1269971)
Supplement: Supplementary file 1 [file DataSheet_1.docx]

Supplement 1. Oncotype Dx according to years.

| Year | Oncotype Dx done | Total patients |
| --- | --- | --- |
| 2010 | 0 | 540 |
| 2011 | 1 | 510 |
| 2013 | 12 | 444 |
| 2014 | 55 | 431 |
| 2015 | 46 | 495 |
| 2016 | 78 | 595 |
| 2017 | 99 | 805 |
| Total | 291 | 4554 |

Supplement 2. Chemotherapy and Oncotype Dx result

|  | Chemotherapy (-) | Chemotherapy (+) |
| --- | --- | --- |
| RS low ( < 15) | 156 | 7 |
| RS intermediate (15-25) | 65 | 11 |
| RS high ( >25 ) | 4 | 48 |

P<0.001

Supplement 3. Disease-free survival according to Oncotype Dx group and chemotherapy.


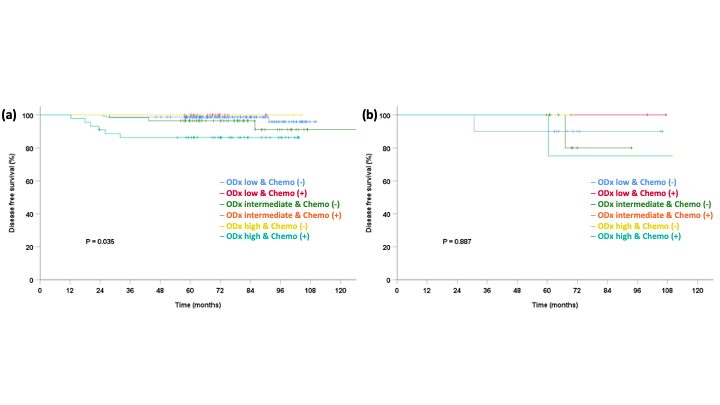


1. LVI negative (b) LVI positive
